# Differences between rural and urban prostate cancer patients

**DOI:** 10.1007/s00345-020-03483-7

**Published:** 2020-11-05

**Authors:** Lara Franziska Stolzenbach, Marina Deuker, Claudia Collà-Ruvolo, Luigi Nocera, Zhe Tian, Tobias Maurer, Derya Tilki, Alberto Briganti, Fred Saad, Vincenzo Mirone, Felix K. H. Chun, Markus Graefen, Pierre I. Karakiewicz

**Affiliations:** 1 Martini-Klinik Prostate Cancer Center, University Hospital Hamburg-Eppendorf, Martinistraße 52, 20246 Hamburg, Germany; 2 Cancer Prognostics and Health Outcomes Unit, Division of Urology, University of Montreal Health Center, Montreal, Quebec Canada; 3 Department of Urology, University Hospital Frankfurt, Frankfurt am Main, Germany; 4 Department of Urology and Division of Experimental Oncology, URI, Urological Research Institute, IRCCS San Raffaele Scientific Institute, Milan, Italy; 5 Department of Neurosciences, Reproductive Sciences and Odontostomatology, Federico II University of Naples, Naples, Italy

**Keywords:** SEER, Localised prostate cancer, Metastatic prostate cancer, Population density, Other cause mortality, North American population

## Abstract

**Background:**

We hypothesized that the residency status (rural area [RA] vs urban clusters [UC] vs urban areas [UA]) affects stage and cancer-specific mortality (CSM) in contemporary newly diagnosed prostate cancer (PCa) patients of all stages, regardless of treatment.

**Methods:**

Newly diagnosed PCa patients with available residency status were abstracted from the Surveillance, Epidemiology, and End Results database (2004–2016). Propensity-score (PS) matching, cumulative incidence plots, multivariate competing-risks regression (CRR) models were used.

**Results:**

Of 531,468 PCa patients of all stages, 6653 (1.3%) resided in RA, 50,932 (9.6%) in UC and 473,883 (89.2%) in UA. No statistically significant or clinically meaningful differences in stage at presentation or CSM were recorded. Conversely, 10-year other cause-mortality (OCM) rates were 27.2% vs 23.7% vs 18.9% (*p* < 0.001) in RA vs UC vs UA patients, respectively. In CRR models, RA (subhazard ratio [SHR] 1.38; *p* < 0.001) and UC (SHR 1.18; *p* < 0.001) were independent predictors for higher OCM relative to UA. These differences remained statistically significant in fully PS-adjusted multivariate CRR models.

**Conclusion:**

RA, and to a lesser extent UC, PCa patients are at higher risk of OCM than UA patients. Higher OCM may indicate shorter life expectancy and should be considered in treatment decision making.

**Electronic supplementary material:**

The online version of this article (10.1007/s00345-020-03483-7) contains supplementary material, which is available to authorized users.

## Introduction

Centres for Disease Control and Prevention (CDC) reported that North Americans living in rural areas are more likely to die from the five leading causes (heart disease, cancer, unintentional injury, chronic lower respiratory disease and stroke) than their urban counterparts. Specifically, in 2014, the number of potentially excess deaths (aged < 80 years) in rural areas of the United States were 25,278 from heart disease, 19,055 from cancer, 12,165 from unintentional injury, 10,676 from chronic lower respiratory disease, and 4108 from stroke [1]. Rural residents have higher rates of cigarette smoking, hypertension, obesity, and physical inactivity during leisure time [2]. Moreover, they are reported to live more frequently in poverty versus urban residents in 2014 (18.1% and 15.1%, respectively) [3]. Similarly, access to health care also differ according to residency status. Specifically, rural residents have less access to health care and lower quality of health care [4, 5].However, few historical reports addressed cancer specific morality (CSM) in prostate cancer patients (PCa) according to residency status [5–8]. All previous reports were based on Australian cohorts and recorded worse CSM in rural PCa patients. However, two recent systematic review, of 45 studies between 1984 and 2016 and of 169 studies between 1998 and 2018, described inconsistent effect of residency status (rural vs urban) on CSM in colorectal, lung, breast and prostate cancers [5, 9].

In consequence, a contemporary North American reassessment of this topic is needed. We addressed this void. Specifically, we tested for differences in CSM and other cause-mortality (OCM), according to residence in urban areas vs urban clusters vs rural areas, a stratification recommended by United States Census Bureau [10]. We hypothesized that rural residence may result in higher CSM in contemporary, newly diagnosed prostate cancer (PCa) patients of all stages.

## Patients and methods

### Study population

The most contemporary Surveillance, Epidemiology, and End Results database (SEER) database samples 34.6% of the United States population and approximates the United States population of demographic composition and cancer incidence [7]. Within the SEER database (2004 − 2016), we identified patients ≥ 18 years-old with histologically confirmed adenocarcinoma of the prostate (International Classification of Disease for Oncology [ICD-O-3] code 8140 site code C61.9) [11]. Cases identified only by autopsy or death certificate, and patients with unavailable residency status were excluded (*n* = 105). These selection criteria yielded 531,468 PCa patients.

### Variable definition

Residency status was defined according to the US Census Bureau definition and consisted of either urban areas (UA) or urban clusters (UC) or rural areas (RA). UA were defined as areas with 50,000 or more residents. UC were defined as areas with at least 2500 but fewer than 50,000 residents. RA, conversely, were defined as all population, housing, and territory not included within UA or UC. Covariates consisted of continuously coded age, year of diagnosis, PSA at diagnosis, biopsy Gleason grade groups (GGG), clinical T stage (cT1, cT2, cT3, cT4), clinical N (cN0, cNX, cN1), M1 stages (M0, MX, M1), and socio-economic status ([SES] 1st quartile, 2nd-3rd-4th- quartiles). According to SEER mortality code, CSM was defined as deaths related to prostate cancer. All other deaths were considered as other-cause mortality (OCM).

### Statistical analyses

Descriptive statistics included frequencies and proportions for categorical variables. Means, medians and ranges were reported for continuously coded variables. Chi-square tested for statistical significance in proportions’ differences. The statistical significance of mean and median differences were tested with t test and Kruskal–Wallis test.

Three analytical steps were performed. First, we tested the effect of residency status (RA vs UC vs UA) on CSM and OCM, in the overall cohort, using cumulative incidence plots and competing-risks regression (CRR) models (Package ‘cmprsk’) [12, 13]. Second, we stratified the patient cohort between non-metastatic T_1-2_ N0 M0 PCa vs locally advanced (T_3-4_ N0 M0) and/or metastatic (T_1-4_ N0/N1 M1) PCa patients. Within those two groups, we compared respectively RA vs UA, RA vs UC and UA vs UC residing patients. In each of the six comparisons, we relied on 1:4 propensity-score (PS) matching for age at diagnosis, year of diagnosis, PSA at diagnosis, biopsy GGG, clinical T stage, N and M stages and treatment type [14, 15]. The propensity matched cohorts were balanced according to all adjustment variables. Third, after 1:4 PS-matching cumulative incidence plots and competing-risks regression (CRR) models were used [12, 13]. Multivariate CRR models accounted for the effect of OCM on CSM and vice versa, to provide the most unbiased estimates, after further adjustment for marital status, race and SES. For all statistical analyses, R software environment for statistical computing and graphics (version 3.4.3) was used. All tests were two sided with a level of significance set at *p* < 0.05 [16].

## Results

### Descriptive characteristics of the study population

Overall, 531,671 PCa patients of all stages were identified (Table 1). Of these, 6653 (1.3%) resided in RA, 50,932 (9.6%) resided in UC and 473,883 (89.2%) resided in UA. Median age was comparable between RA, UC, and UA (67 vs 66 vs 65 years). Married patients were most frequently recorded in RA (69.4%), followed by UC (68.5%), followed by UA (66.4%). Caucasian race was most frequently recorded in RA (89.0%), followed by UC (82.2%), followed by UA (66.6%).

**Table 1:** Descriptive characteristics of prostate cancer patients (*n* = 531,468) of all stages stratified between rural areas, urban clusters and urban areas according to the United States Census Bureau urban–rural classification identified within the Surveillance, Epidemiology, and End Results database between 2004 and 2016

		Overall cohort 531,468	Rural areas 6653 (1.3%)	Urban clusters 50,932 (9.6%)	Urban areas 473,883 (89.2%)	*p* value
Age at diagnosis, years	Median	65	67	66	65	< 0.001
	IQR	59–72	61–73	60–72	59–72	
PSA, ng/ml	Median	6.5	7.2	7.1	6.5	< 0.001
	IQR	4.8–10.6	5.1–12.6	5–12	4.7–10.5	
Biopsy Gleason Grade Groups, *n* (%)	I	209,437 (39.4)	2377 (35.7)	18,770 (36.9)	188,290 (39.7)	< 0.001
	II	137,886 (25.9)	1694 (25.5)	13,107 (25.7)	123,085 (26)	
	III	60,171 (11.3)	810 (12.2)	5945 (11.7)	53,416 (11.3)	
	IV	46,770 (8.8)	616 (9.3)	4644 (9.1)	41,510 (8.8)	
	V	40,679 (7.7)	665 (10.0)	4556 (8.9)	35,458 (7.5)	
	Unknown GS	36,525 (6.9)	491 (7.4)	3910 (7.7)	32,124 (6.8)	
Clinical T-Stage, *n* (%)	cT1	324,809 (61.1)	3773 (56.7)	29,369 (57.7)	291,667 (61.5)	< 0.001
	cT2	163,982 (30.9)	2344 (35.2)	17,475 (34.3)	144,163 (30.4)	
	cT3	14,074 (2.6)	206 (3.1)	1557 (3.1)	12,311 (2.6)	
	cT4	4699 (0.9)	72 (1.1)	520 (1.0)	4107 (0.9)	
	Unknown	23,904 (4.5)	258 (3.9)	2011 (3.9)	21,635 (4.6)	
Nodal Stage, *n* (%)	cN0	493,095 (92.8)	6160 (92.6)	47,161 (92.6)	439,774 (92.8)	< 0.001
	cN1	15,047 (2.8)	200 (3.0)	1412 (2.8)	13,435 (2.8)	
	cNX	23,326 (4.4)	293 (4.4)	2359 (4.6)	20,674 (4.4)	
M-Stage, *n* (%)	M0	495,539 (93.2)	6151 (92.5)	47,136 (92.5)	442,252 (93.3)	< 0.001
	M1	22,386 (4.2)	331 (5.0)	2437 (4.8)	19,618 (4.1)	
	MX	13,543 (2.5)	171 (2.6)	1359 (2.7)	12,013 (2.5)	
Treatment, *n* (%)	No local treatment	139,998 (26.3)	1732 (26.0)	13,821 (27.1)	124,445 (26.3)	< 0.001
	Radical prostatectomy	193,124 (36.3)	2341 (35.2)	17,130 (33.6)	173,653 (36.6)	
	Radiotherapy	182,320 (34.3)	2352 (35.4)	18,080 (35.5)	161,888 (34.2)	
	Unknown treatment	16,026 (3.0)	228 (3.4)	1901 (3.7)	13,897 (2.9)	
Marital Status, *n* (%)	Married	354,255 (66.7)	4620 (69.4)	34,904 (68.5)	314,731 (66.4)	< 0.001
	Unmarried	116,744 (22)	1300 (19.5)	10,763 (21.1)	104,681 (22.1)	
	Unknown	60,469 (11.4)	733 (11)	5265 (10.3)	54,471 (11.5)	
Race, *n* (%)	Caucasian	363,190 (68.3)	5922 (89.0)	41,860 (82.2)	315,408 (66.6)	< 0.001
	African-American	81,904 (15.4)	580 (8.7)	5798 (11.4)	75,526 (15.9)	
	Hispanic	48,812 (9.2)	87 (1.3)	1791 (3.5)	46,934 (9.9)	
	Native	1659 (0.3)	12 (0.2)	385 (0.8)	1262 (0.3)	
	Asian	26,008 (4.9)	6 (0.1)	659 (1.3)	25,343 (5.3)	
	Unknown	9895 (1.9)	46 (0.7)	439 (0.9)	9410 (2)	
SEER registry, *n* (%)	Atlanta	20,779 (3.9)	0 (0)	0 (0)	20,779 (4.4)	< 0.001
	California	112,687 (21.2)	308 (4.6)	6288 (12.3)	106,091 (22.4)	
	Connecticut	25,196 (4.7)	0 (0)	1536 (3)	23,660 (5)	
	Detroit	30,954 (5.8)	0 (0)	0 (0)	30,954 (6.5)	
	Greater Georgia	39,484 (7.4)	1498 (22.5)	9636 (18.9)	28,350 (6)	
	Hawaii	7742 (1.5)	0 (0)	1149 (2.3)	6593 (1.4)	
	Iowa	20,759 (3.9)	1509 (22.7)	9023 (17.7)	10,227 (2.2)	
	Kentucky	25,280 (4.8)	2473 (37.2)	8964 (17.6)	13,843 (2.9)	
	Los Angeles	48,130 (9.1)	0 (0)	0 (0)	48,130 (10.2)	
	Louisiana	33,272 (6.3)	310 (4.7)	5276 (10.4)	27,686 (5.8)	
	New Jersey	64,444 (12.1)	0 (0)	0 (0)	64,444 (13.6)	
	New Mexico	11,167 (2.1)	143 (2.1)	3457 (6.8)	7567 (1.6)	
	Rural Georgia	1200 (0.2)	82 (1.2)	840 (1.6)	278 (0.1)	
	San Francisco-Oakland	29,101 (5.5)	0 (0)	0 (0)	29,101 (6.1)	
	San Jose-Monterey	15,834 (3.0)	0 (0)	0 (0)	15,834 (3.3)	
	Seattle (Puget Sound)	29,949 (5.6)	227 (3.4)	2940 (5.8)	26,782 (5.7)	
	Utah	15,490 (2.9)	103 (1.5)	1823 (3.6)	13,564 (2.9)	
Socio economic status, *n*(%)	1 quartile	146,722 (27.6)	2012 (30.2)	18,960 (37.2)	125,750 (26.5)	< 0.001
	2–3-4 quartile	384,746 (72.4)	4641 (69.8)	31,972 (62.8)	348,133 (73.5)	

PSA at diagnosis (median) was comparable between the examined groups and showed absolute differences of 0.1–0.7 ng/ml (Table 1). Similarly, in RA vs UC vs UA, N0-stage was recorded in 92.6% vs 92.6% vs 92.8% patients and M0-stage was recorded in 92.5%, vs 92.5% vs 93.3% patients, respectively. Conversely, clinical T1 stage was less prevalent in RA (56.7%), vs UC (57.7%),vs UA (61.5%), as well as biopsy GGG I was less prevalent in RA (35.7%), vs UC (36.9%), vs UA (39.7%). Rates of all treatment types were comparable and showed absolute differences of 0.1–2.0% (Table 1).

Temporal trend analyses demonstrated, increasing rates of GGG IV or V (Supplementary Fig. 1b), clinical T_3-4_, N1 and M1 stages (Supplementary Fig. 1c–d) in all three examined groups (RA, UC and UA) (*p* < 0.05). Exceptions consisted of median PSA at diagnosis in RA and UA patients (Supplementary Fig. 1a) that did not change over time (*p* > 0.05).

In six separate multivariate logistic regression analyses, predicting (1) locally advanced tumor stage (T_3-4_) and/or node positive stage (N_1_), (2) metastatic stage (M_1_), (3) biopsy GGG IV-V, (4) treatment with RP (5) treatment with RT and (6) no treatment, RA residency status did not predict the examined outcomes (Supplementary Table 1) with one exception, RA predicted biopsy GGG IV-V (OR 1.11; *p* = 0.003). Moreover, UC residency status predicted higher rates of radiotherapy (OR 1.03, *p* = 0.003) and no local treatment (OR 1.03; *p* = 0.02) and lower rates of radical prostatectomy (OR 0.89; *p* < 0.001), when referenced to UA residency status.

### Unadjusted and unmatched survival analyses: overall cohort

**Fig. 1:**
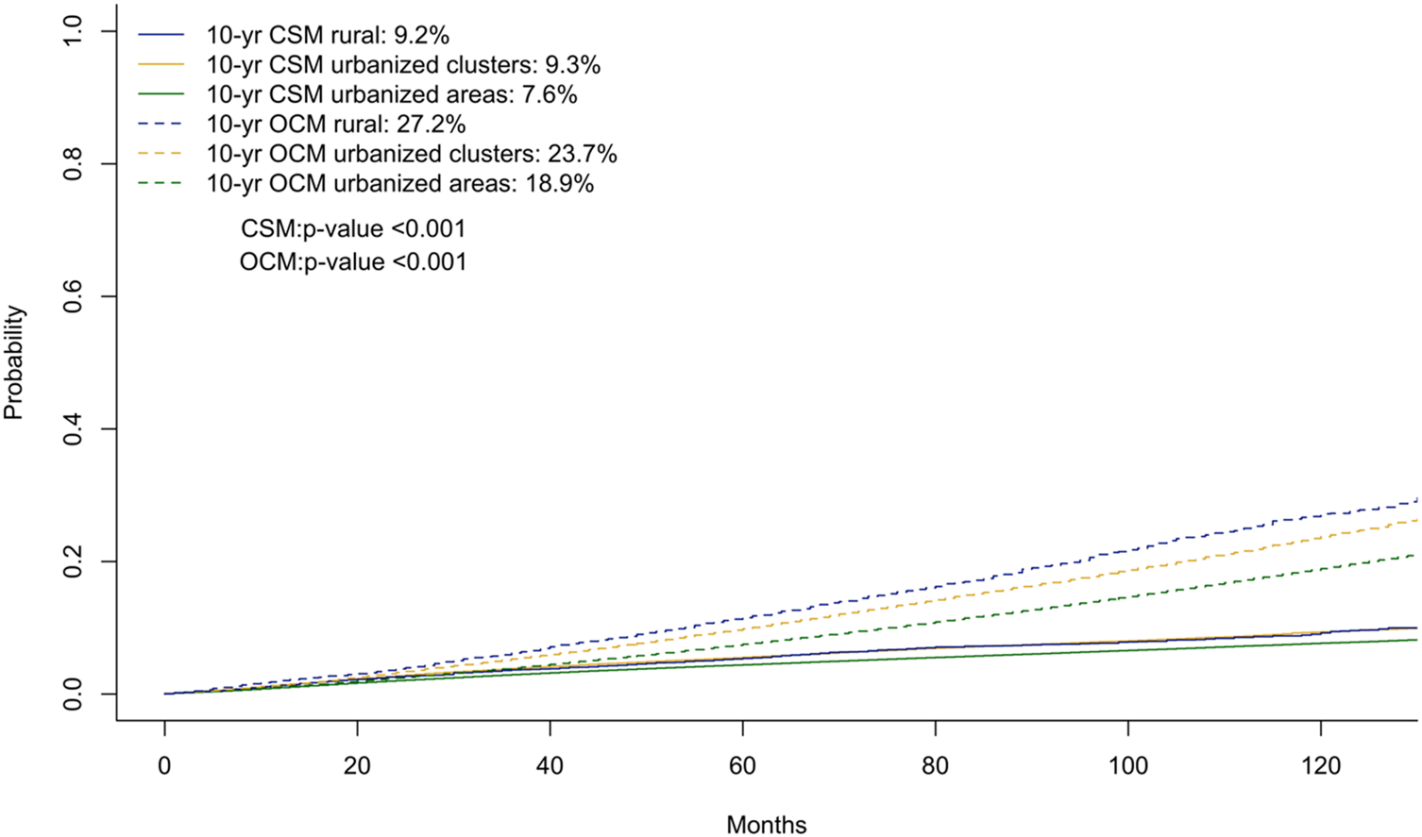
Cumulative incidence plot of cancer specific mortality (CSM) and other cause mortality (OCM) in prostate cancer patients of all stages, stratified between rural areas, urban clusters and urban areas according to the United States Census Bureau urban–rural classification

In the first part of survival analyses, we relied on cumulative incidence plots to test for differences in CSM and OCM between RA vs UC vs UA (Fig. 1). Ten-year CSM rates according to RA vs UC vs UA status were 9.2% vs 9.3% vs 7.6% (*p* < 0.01), which resulted in subhazard ratios (SHR) for CSM of 0.95 (*p* = 0.4) in RA and of 1.07 (95% CI 1.03–1.12; *p* < 0.001) in UC, relative to UA. The corresponding 10-year OCM rates according to RA vs UC vs UA status were 27.2% vs 23.7% vs 18.9% (*p* < 0.001), which resulted in SHR for OCM of 1.38 (*p* < 0.001) in RA and of 1.18 (*p* < 0.001) in UC, relative to UA.

### Stage stratified and propensity-score adjusted survival analyses

**Fig. 2:**
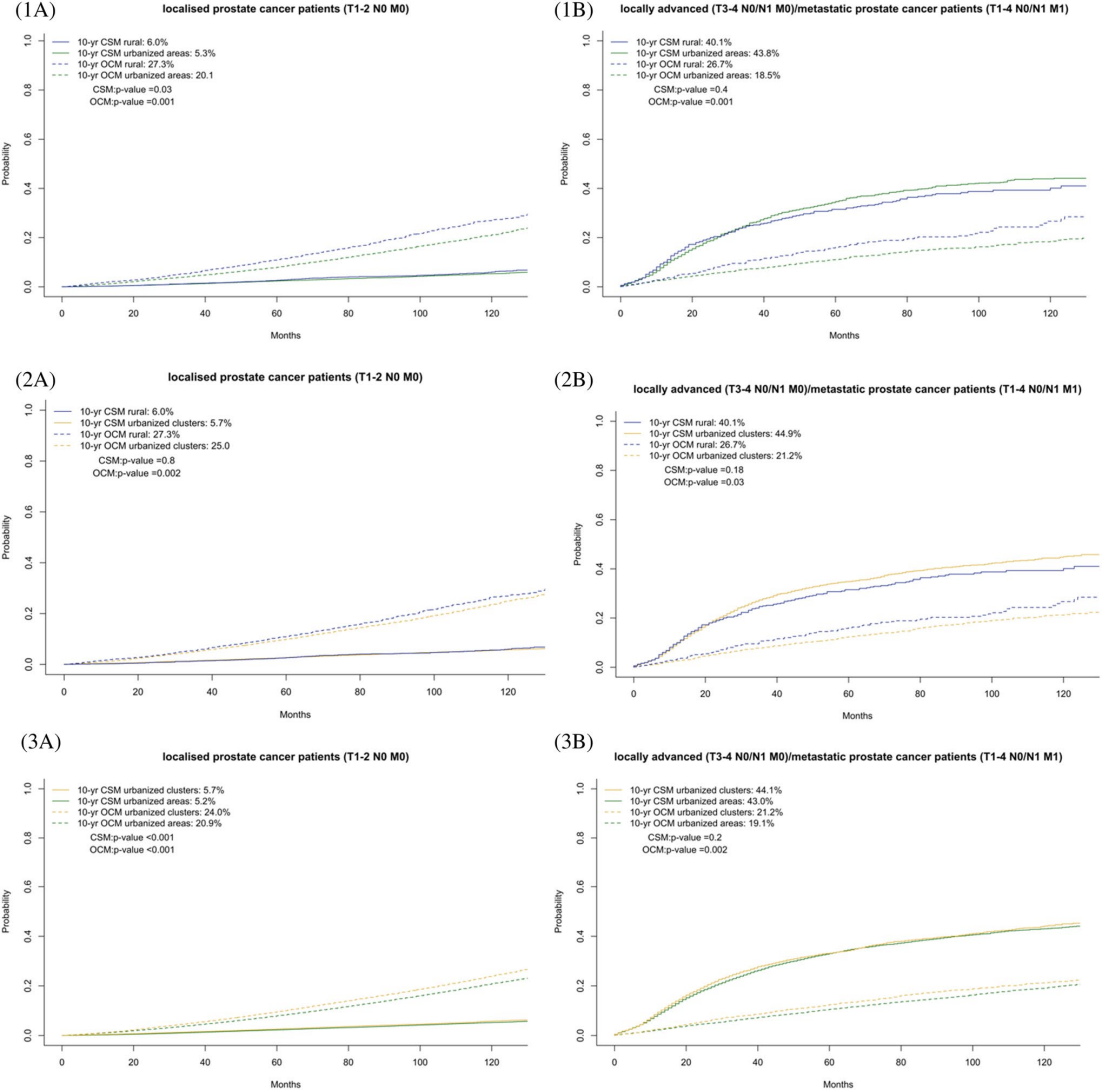
**a** Cumulative incidence plot of cancer specific mortality (CSM) and other cause mortality (OCM) in stage. T1-2N0 M0 prostate cancer patients after 1:4 propensity score matching of (1) rural areas to urban areas (*n* = 29,150) or (2) rural areas to urban clusters (*n* = 29,150) and of (3) urban clusters to urban areas (*n* = 223,545) residing patients. **b** Cumulative incidence plot of CSM and OCM in stage T3-4 N0 M0 or T1-4 N0/N1 and/ or M1 prostate cancer patients after 1:4 propensity score matching of (1) rural areas to urban areas (*n* = 4115) or (2) rural areas to urban clusters (*n* = 4115) and of (3) urban clusters to urban areas (*n* = 31,115) residing patients

In the second part of survival analyses, we performed more stringent testing that relied on stratification according to stage. Moreover, 1:4 PS-matching was applied between the examined groups. Here one RA patient was matched with either four UC or four UA patients. Similarly, one UC patient was matched with four UA patients. Since PS-matching only applies to comparisons between 2 groups, we performed 3 comparisons: (1). RA vs UA, (2). RA vs UC and (3) UC vs UA. In addition to cumulative incidence plots (Fig. 2), multivariate CRRs were fitted that adjusted for residual confounding covariates (Table 2).

**Table 2:** Multivariate competing risks regression analyses testing the effect of rural areas vs urban clusters vs urban areas according to the United States Census Bureau’s urban–rural classification on cancer-specific mortality (CSM) and other-cause mortality (OCM) in prostate cancer patients of all stages within the Surveillance, Epidemiology and End Results (2004–2016) database

Variable	Cancer-specific mortality		Other cause mortality	
	Multivariate HR (95% CI)	*p* value	Multivariate HR (95% CI)	*p* value
A. Overall cohort (*n* = 531,671)				
Urban areas	1.00 (Ref.)	–	1.00 (Ref.)	–
Urban clusters	1.07 (1.03–1.12)	0.001	1.18 (1.15–1.21)	< 0.001
Rural areas	0.95 (0.85–1.07)	0.4	1.38 (1.3–1.47)	< 0.001
B. After 1:4 PS-matching of rural (*n* = 5830) to urban areas (*n* = 23,320) T1-2 N0 M0 patients				
Urban areas	1.00 (Ref.)	–	1.00 (Ref.)	–
Rural areas	1.12 (0.96–1.3)	0.16	1.44 (1.33–1.54)	< 0.001
C. After 1:4 PS-matching of rural (*n* = 823) to urban areas (*n* = 3292) T3-4 N0/1 and M0 patients				
Urban areas	1.00 (Ref.)	–	1.00 (Ref.)	–
Rural areas	0.91 (0.76–1.08)	0.2	1.53 (1.22–1.93)	< 0.001
D. After 1:4 PS-matching of rural (*n* = 5830) to urban clusters (*n* = 23,320) T1-2 N0 M0 patients				
Urban clusters	1.00 (Ref.)	–	1.00 (Ref.)	–
Rural areas	1.0 (0.86–1.16)	0.9	1.11 (1.04–1.2)	0.002
E. After 1:4 PS-matching of rural (*n* = 823) to urban cluster (*n* = 3292) T3-4 N0/1 and M0 patients				
Urban clusters	1.00 (Ref.)	–	1.00 (Ref.)	–
Rural areas	0.84 (0.72–0.99)	0.04	1.34 (1.08–1.66)	0.001
F. After 1:4 PS-matching of urban cluster (*n* = 44,709) to urban areas (*n* = 178,836) T1-2 N0 M0 patients				
Urban areas	1.00 (Ref.)	–	1.00 (Ref.)	–
Urban clusters	1.13 (1.07–1.2)	< 0.001	1.23 (1.2–1.27)	< 0.001
G. After 1:4 PS-matching of urban cluster (*n* = 6223) to urban areas (*n* = 24,892) T3-4 N0/1 and M0 patients				
Urban areas	1.00 (Ref.)	–	1.00 (Ref.)	–
Urban clusters	1.02 (0.96–1.08)	0.4	1.15 (1.06–1.26)	0.001

### *Non-metastatic T*_*1-2*_* N0 M0 prostate cancer patients*

In T_1-2_ N0 M0 PCa patients, 10-year OCM rates were 27.3 vs 20.1%, 27.3 vs 25.0% and 24.0 vs 20.9%, in respectively RA vs UA, RA vs UC and UC vs UA (all *p* < 0.001). In CRR models, OCM rate was 1.44- (*p* < 0.001) and 1.11- (*p* = 0.002) fold higher in respectively RA vs UA and RA vs UC patients. Similarly, OCM rate was 1.23-fold higher in UC vs UA patients (*p* < 0.001).

The corresponding 10-years CSM rates were 6.0 vs 5.3% (*p* = 0.03), 6.0 vs 5.7% (*p* = 0.8) and 5.7 vs 5.2% (*p* < 0.001), in respectively RA vs UA, RA vs UC and UC vs UA. In CRR models, no statistically significant or clinically meaningful differences were recorded in the corresponding CSM rates (all *p* > 0.05).

### *Locally advanced T*_*3-4*_* N0 M0 or metastatic T*_*1-4*_* N0/N1 M1 prostate cancer patients*

In T_3-4_ N0 M0 and T_1-4_ N0/N1 M1, 10-year OCM rates were 26.6 vs 19.2%, 26.6 vs 21.2% and 21.2 vs 19.1%, in respectively RA vs UA, RA vs UC and UC vs UA (all *p* < 0.05). In multivariate CRR models, OCM rate was 1.53- (*p* < 0.001), 1.34- (*p* = 0.001) and 1.15- (*p* = 0.001) fold higher in respectively RA vs UA, RA vs UC and UA vs UC patients (all *p* < 0.01).

The corresponding 10-year CSM rates were 40.1 vs 43.8% (*p* = 0.4), 40.1 vs 44.9% (*p* = 0.2) and 44.1 vs 43.0% (*p* = 0.2), in respectively RA vs UA, RA vs UC and UC vs UA. In CRR models, no statistically significant or clinically meaningful differences were recorded in the corresponding CSM rates (all *p* > 0.05).

## Discussion

We hypothesized that residency status (rural areas vs urbanized clusters vs urbanized areas), defined according to the United States Census Bureau recommendations, affects stage and CSM in contemporary newly diagnosed prostate cancer (PCa) patients. Our analyses resulted in several important observations.

First, of all PCa patients in our analyses, only 1.3% accounted for RA residency status. Conversely, 9.6% were recorded in UC and 89.2% in UA residential areas, respectively. These rates differ substantially from the officially reported composition of the US according to the US Census Bureau [10], in year 2010, there were 486 UA and 3087 UC in the United States. UA accounted for 71.2% of the US population, while 9.5% resided in UC. Conversely, 19.3% resided in RA. Taking into account these major differences in the composition of the US population and the composition of the SEER database, it has to be stated that the SEER database does not reflect the US in terms of urban vs rural residency status. Rural regions of the US population are severely underrepresented in the SEER database. This fact is attributable to the composition of the SEER registries, that encompass mainly metropolitan regions [11]. In consequence, it is difficult to analyze the effect of rural residency status within the SEER database, due to excessively small number of RA observations. Ideally, future iterations of the SEER database should oversample rural areas, to better reflect the rural composition of the US.

Second, we recorded marginal differences in stage of presentation and in treatment rates, within the three examined groups. The most pronounced differences were recorded in clinical T stage and biopsy GGG. RA residing patients more frequently harboured GGG IV or V (19.3%) and T3/T4 -stages (4.2%), relative to UC (18.0, 4.2%) and UA (16.3, 3.5%) residing patients. These observations were in accordance with two previous historical studies, conducted in Australian cohorts [6, 7]. For example, Yu et al. (*n* = 68,686; 1982–2004) reported higher rates of advanced PCa in RA patients relative to UA patients (10.8% vs 9.9%) [7]. However, we recorded in the most contemporary years only subtle differences in stage of presentation. These observations are encouraging, because it indicates that current efforts aimed at reducing the stage, grade and PSA disadvantage gap between rural and urban residents.

Third, in survival analyses, RA and UC patients exhibited higher rates of OCM than UA patients. Even after strict adjustments for patient and tumor characteristics and additionally for CSM, RA and UC residence status were independent predictors of higher OCM in non-metastatic PCa, as well as in locally advanced or metastatic prostate cancer relative to UA. To the best of our knowledge, we are first to describe those discrepancies of OCM in PCa patients. Those differences may affect life expectancy. To date, life expectancy calculators recommended by guidelines for PCa, do not account for residency status [17]. For example, the United States Social Security life expectancy calculator does not account for 1.44-fold higher risk for OCM in RA PCa patients. Moreover, the Centers for Disease Control and Prevention also reported that rural residents were more likely to die of the five leading causes (heart, cancer, unintentional injuries, chronic respiratory disease and stroke) than their urban counterparts [1]. However, urologists should be aware of those differences in OCM rates, when treatment considerations were made because life expectancy is an important factor in North American and European guidelines recommendations.

Fourth, unlike for OCM, RA residing patients were not at higher risk of prostate cancer mortality. A marginally, albeit statistically significantly higher CSM rate was recorded in UC PCa patients relative to UA (SHR1.07; *p* < 0.05). The clinical significance of such marginally higher rate is of unknown importance. These observations are in disagreement with previous historical reports, where RA was associated with higher CSM relative to UA, hazard ratios ranged from 1.31 (95% CI 1.22–1.41) to 1.61 (95% CI 1.46–1.77) [6–8]. However, these studies reported on Australian data and on historical patient cohorts.

Taken together, our study demonstrated several noteworthy findings. After most stringent matching and adjustment for CSM, we recorded important differences in OCM, according to rural vs urban residency status. Specifically, RA and UC residing patients exhibited statistically significantly and clinically meaningful higher OCM. The presence of these prognostically unfavorable mortality differences in RA and UC relative to UA, requires prompt recognition regarding health policy and should also be taken into account in treatment considerations. Conversely, we did not find important differences between rural and urban PCa patients regarding CSM.

Despite multiple novel and important observations, several limitations may be applicable to our study. First and foremost, the number of patients in RA residency status was low and did not allow a proportional representation of the composition of the US population. Due to this limitation, no further subgroup analyses addressing race could be performed. In consequence, future analyses of other databases might investigate this topic. Moreover, the retrospective, population-based nature of the SEER database, did not allow us to control for important covariates such as comorbidities that are crucial for survival analyses. Nonetheless, we relied on OCM, which directly accounts for most important comorbidities, namely those that result in death. These limitations related to the retrospective, population-based nature of the SEER database, apply to this, as well as to other similar analyses that were based on the SEER database or on other similar large scale data repositories, such as National Cancer Data Base, National Inpatient Sample or National Surgical Quality Improvement Program.

## Conclusion

RA, and to a lesser extent UC, PCa patients are at higher risk of other cause mortality than UA patients. Higher OCM may indicate shorter life expectancy and should be considered in treatment decision making.

## Electronic supplementary material

Below is the link to the electronic supplementary material.

Supplementary Fig.1 Estimated annual percentage change (EAPC) plots depicting the distribution of annual rates (2004-2016) between prostate cancer patients of rural areas, urban clusters and urban areas according to PSA at initial diagnosis (A), biopsy Gleason grade group (B) and stage (C) in the overall cohort (n=531,468) (DOCX 2896 kb)

Supplementary Table 1 Six separate logistic regression models predicting predicting A) locally advanced tumor stage (T3-4) and/or node positive stage (N1), B) metastatic stage (M1), C) biopsy GGG IV-V, D) treatment with RP E) treatment with RT and F) no treatment according to residency status rural area vs. urban clusters, reference: urbanized areas). Abbreviations: OR=Odds ratio, CI=confidence interval, RP=radical prostatectomy, RT=radiotherapy (DOCX 17 kb)
