# Supplementary material for: Differences between rural and urban prostate cancer patients
Source: World J Urol. 2020 Nov 5;39(7):2507–14. doi: 10.1007/s00345-020-03483-7 (PMC8332582; doi:10.1007/s00345-020-03483-7)

**Supplementary Figure 1:**

Estimated annual percentage change (EAPC) plots depicting the distribution of annual rates (2004-2016)

between prostate cancer patients of rural areas, urban clusters and urban areas according to PSA at initial diagnosis (A), biopsy Gleason grade group (B) and stage (C) in the overall cohort (n=531,468).


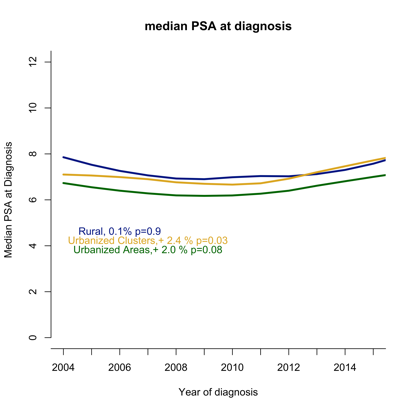


A

D

C

B


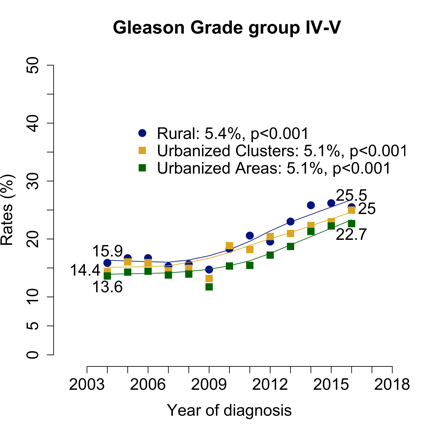

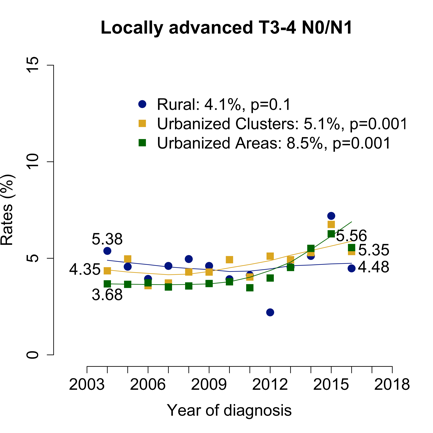

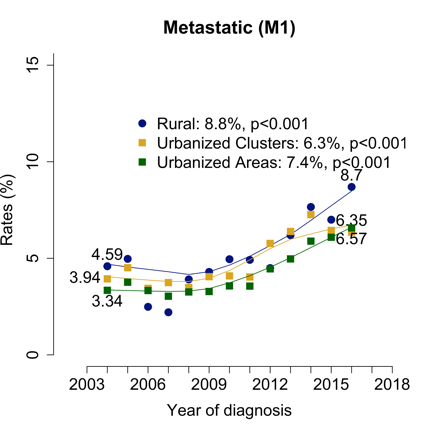

Supplement: Supplementary file 1 — Supplementary Fig.1 Estimated annual percentage change (EAPC) plots depicting the distribution of annual rates (2004-2016) between prostate cancer patients of rural areas, urban clusters and urban areas according to PSA at initial diagnosis (A), biopsy Gleason grade group (B) and stage (C) in the overall cohort (n=531,468) (DOCX 2896 kb) [file 345_2020_3483_MOESM1_ESM.docx]
