# Supplementary material for: Differences between rural and urban prostate cancer patients
Source: World J Urol. 2020 Nov 5;39(7):2507–14. doi: 10.1007/s00345-020-03483-7 (PMC8332582; doi:10.1007/s00345-020-03483-7)
Supplement: Supplementary file 2 — Supplementary Table 1 Six separate logistic regression models predicting predicting A) locally advanced tumor stage (T3-4) and/or node positive stage (N1), B) metastatic stage (M1), C) biopsy GGG IV-V, D) treatment with RP E) treatment with RT and F) no treatment according to residency status rural area vs. urban clusters, reference: urbanized areas). Abbreviations: OR=Odds ratio, CI=confidence interval, RP=radical prostatectomy, RT=radiotherapy (DOCX 17 kb) [file 345_2020_3483_MOESM2_ESM.docx]

**Supplementary Table 1.**

Six separate logistic regression models predicting predicting A) locally advanced tumor stage (T_3-4_) and/or node positive stage (N_1_), B) metastatic stage (M_1_), C) biopsy GGG IV-V, D) treatment with RP E) treatment with RT and F) no treatment according to residency status rural area vs. urban clusters, reference: urbanized areas).

Abbreviations: OR=Odds ratio, CI=confidence interval, RP=radical prostatectomy, RT=radiotherapy

|  | **A. Predictors of advanced**  **stage (T_3-4/_ N1)** | | **B. Predictors of metastatic stage M_1_** | | **C. Predictors of biopsy Gleason grade group IV-V** | |
| --- | --- | --- | --- | --- | --- | --- |
|  | **OR (95% CI)** | **p-value** | **OR (95% CI)** | **p-value** | **OR (95% CI)** | **p-value** |
| **Urbanized Areas (Ref.)** | 1 | - | 1 | - | 1 | - |
| **Rural Areas** | 0.88 (0.77-1.01) | 0.06 | 0.99 (0.94-1.06) | 0.8 | 1.11 (1.04-1.19) | 0.003 |
| **Urban Clusters** | 0.98 (0.93-1.03) | 0.43 | 0.90 (0.87-0.92) | **0.01** | 1.04 (1.01-1.07) | 0.003 |

|  | **D. Predictors of treatment with RP** | | **E. Predictors of treatment with RT** | | **F. Predictors of no local treatment** | |
| --- | --- | --- | --- | --- | --- | --- |
|  | **OR (95% CI)** | **p-value** | **OR (95% CI)** | **p-value** | **OR (95% CI)** | **p-value** |
| **Urbanized Areas (Ref.)** | 1 | - | 1 | - | 1 | - |
| **Rural Areas** | 0.99 (0.94-1.07) | 0.81 | 1.02 (0.96-1.07) | 0.49 | 0.96 (0.90-1.02) | 0.26 |
| **Urban Clusters** | 0.89 (0.87-0.92) | **<0.001** | 1.03 (1.03-1.05) | **0.003** | 1.03 (1.01-1.05) | **0.02** |
